# Piezosurgery in Third Molar Extractions: A Systematic Review

**DOI:** 10.3390/jpm14121158

**Published:** 2024-12-19

**Authors:** Antonio Mancini, Angelo Michele Inchingolo, Fabrizio Chirico, Giuseppe Colella, Fabio Piras, Valeria Colonna, Pierluigi Marotti, Claudio Carone, Alessio Danilo Inchingolo, Francesco Inchingolo, Gianna Dipalma

**Affiliations:** 1 Department of Interdisciplinary Medicine, University of Bari “Aldo Moro”, 70124 Bari, Italy; antonio.mancini@uniba.it (A.M.); a.inchingolo3@studenti.uniba.it (A.M.I.); valeria.colonna@uniba.it (V.C.); pierluigi.marotti@uniba.it (P.M.); claudio.carone@uniba.it (C.C.); a.inchingolo1@studenti.uniba.it (A.D.I.); gianna.dipalma@uniba.it (G.D.); 2 Multidisciplinary Department of Medical-Surgical and Dental Specialties, Oral and Maxillofacial Surgery Unit, University of Campania “Luigi Vanvitelli”, 80138 Naples, Italy; fabrizio.chirico@policliniconapoli.it (F.C.); giuseppe.colella@unicampania.it (G.C.)

**Keywords:** piezosurgery, oral surgery, tooth extraction, ultrasonic vibrations, bone healing, postoperative outcomes, third molar extraction

## Abstract

**Background**: The aim of this systematic review was to evaluate the clinical efficacy, benefits, and limitations of piezosurgery in tooth extractions compared to conventional methods. Piezosurgery has emerged as a minimally invasive alternative, promoting better preservation of soft tissues and bone structures. Understanding its impact on postoperative outcomes such as pain, swelling, trismus, and bone healing is critical for its application in oral surgery; **Materials and Methods:** We restricted our search to English-language articles published between 1 January 2004 and 28 August 2024, in PubMed, Scopus, and Web of Science. The Boolean search keywords “piezosurgery AND tooth extraction” were used. **Results:** A total of 983 articles were identified, and after duplicates were removed, 766 studies were screened. Following the application of inclusion and exclusion criteria, seven articles were selected for qualitative analysis. **Conclusions:** The literature suggests that piezosurgery offers advantages, such as reduced postoperative pain, swelling, and trismus, despite longer surgical times compared to conventional methods. While piezosurgery shows promise for improved patient comfort and soft tissue preservation, further research is required to validate its long-term impact on bone healing and regeneration.

## 1. Introduction

One of the most common procedures in oral and maxillofacial surgery is the extraction of teeth. In the past, mechanical tools like elevators and forceps have been used to remove teeth, which frequently results in significant stress to both soft and hard structures [1,2,3,4,5]. Conventional extraction methods are generally successful, but they can present a variety of difficulties, such as significant bone loss, harm to neighboring tissues, protracted healing periods, and postoperative discomfort [6,7,8,9,10]. The need for less invasive dental procedures that maintain adjacent anatomical structures while simultaneously improving patient comfort and healing results is growing in the field of dentistry [11,12,13,14].

As a recent innovation in dental technology, piezosurgery presents a fresh method of tissue handling [15,16,17,18,19,20,21]. Piezosurgery is a technique that was developed in the early 2000s that uses carefully regulated ultrasonic vibrations to minimize damage to adjacent soft tissues like mucosa, blood vessels, and nerves, while precisely cutting hard structures like bone [22,23,24,25,26]. The precision of piezosurgery enables a more focused and non-traumatic surgical intervention than is possible with typical rotational or mechanical devices, which run the risk of unintentionally damaging both soft tissue and bone [27,28,29,30,31,32]. Because of this feature, piezosurgery has emerged as a viable substitute for a series of oral surgery operations, such as sinus augmentation, implant site preparation, and, more recently, dental extractions [33,34,35,36,37].

Given its potential to enhance patient outcomes, there is growing interest among clinicians and researchers in the application of piezosurgery for tooth extractions [38,39,40,41,42]. Several studies suggest that piezosurgery can reduce intraoperative bleeding, postoperative pain and swelling, and lower the risk of complications such as periodontal and alveolar bone loss [43,44,45,46]. Additionally, preserving bone architecture is crucial for patients who may require future dental rehabilitation, such as implant placement [47,48,49]. By minimizing alveolar bone loss following extraction, piezosurgery may improve long-term outcomes for such restorative treatments [50,51,52,53].

However, despite these advantages, piezosurgery is not without limitations [54,55,56,57,58]. The procedure typically requires more time than traditional methods, potentially leading to longer chairside durations [59,60,61,62,63]. Moreover, the high cost of specialized equipment may limit its accessibility in certain clinical settings [64,65,66,67,68]. There is also an ongoing debate regarding the learning curve associated with this technology and its efficacy in complex extraction cases, such as those involving impacted teeth or ankylosed roots [69,70,71,72].

This article aims to critically assess the use of piezosurgery in dental extractions by comparing it to traditional techniques [73,74,75,76,77]. It will explore both the benefits and limitations of piezosurgery based on existing research, with a focus on its clinical applications and implications for dental practice [78,79,80,81,82]. Additionally, this review will highlight areas where further research is needed, helping to evaluate whether piezosurgery can serve as a viable alternative to conventional extraction methods in modern dentistry [83,84,85,86,87].

## 2. Materials and Methods

### 2.1. Protocol and Registration

This systematic review was conducted according to Preferred Reporting Items for Systematic Reviews and Meta-Analyses (PRISMA) [88]. The review protocol was registered at The International Prospective Register of Systematic Reviews (PROSPERO) under the ID: CRD42024594374.

### 2.2. Search Processing

A search on PubMed, Scopus, and Web of Science was performed to find papers that matched the topic of application of piezosurgery in tooth extractions, dating from 1 January 2004 to 28 August 2024, in English. The search strategy used the Boolean keywords: “piezosurgery” AND “tooth extraction” (Table 1).

### 2.3. Inclusion Criteria

The following inclusion criteria were considered: (1) studies that investigated the application of piezosurgery in tooth extractions; (2) randomized clinical trials, (3) English-language articles, and (4) full-text articles.

Papers that did not match the above criteria were excluded.

The review was conducted using the PICOS criteria:

• Participants: both male and female, without pathologies or syndromes, with the necessity of surgical treatment.
• Interventions: application of piezosurgery in third molar extractions.
• Comparisons: control group.
• Outcomes: surgical time, postoperative pain and recovery, trismus and mouth opening, swelling and edema, bone healing and density, bone regeneration.
• Study: randomized clinical trials.

### 2.4. Exclusion Criteria

The exclusion criteria were as follows: (1) animal studies; (2) in vitro studies; (3) off-topic studies; (4) reviews, retrospective studies, case series, case reports, letters, or comments; (5) not English-language studies.

### 2.5. Data Processing

Three reviewers (P.M., V.C. and C.C.) independently consulted the databases to collect the studies and rated their quality based on selection criteria. The selected articles were downloaded in Zotero (Version 6.0.15). Any divergence between the three authors was settled by a discussion with a senior reviewer (F.I.).

### 2.6. Quality Assessment

The quality of the included papers was assessed by two reviewers, F.P. and V.C., using the ROBINS-I tool developed to assess the risk of bias in the results of randomized studies that compare the health effects of two or more interventions. Seven points were evaluated, and each was assigned a degree of bias. A third reviewer (F.I.) was consulted in the event of a disagreement until an agreement was reached.

The question in the domains evaluated in the ROBINS encompasses the following:

- Bias due to confounding;
- Bias arising from the measurement of exposure;
- Bias in the selection of participants in the study;
- Bias due to post-exposure intervention;
- Bias due to missing data;
- Bias arising from the measurement of the outcome;
- Bias in the selection of the reported results, dating from 1 January 2004 to 28 August 2024 and published in in English.

## 3. Results

### 3.1. Study Selection and Characteristics

**Figure 1:**
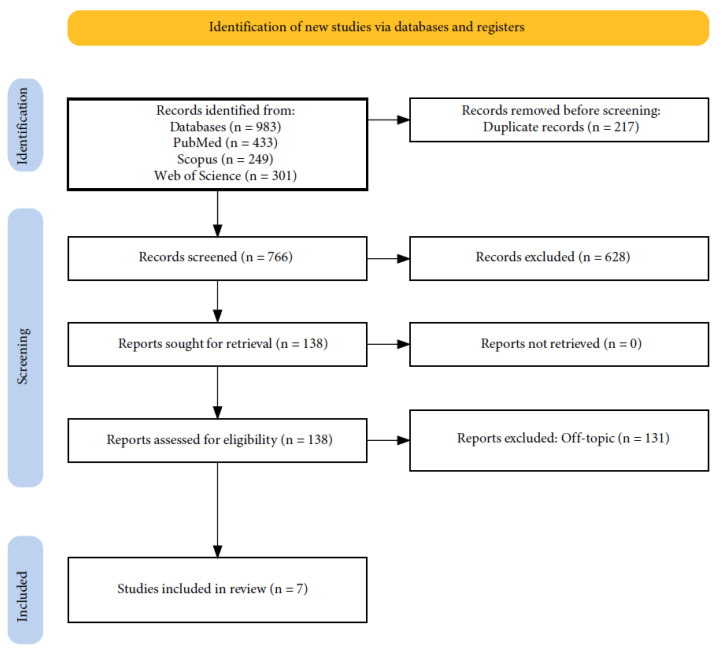
Below are the PRISMA flow diagram and indicators of database search.

The electronic database search identified a total of 983 articles (Scopus N = 249, PubMed N = 433, Web of Science N = 301), and no articles were included through the hand search. After the deletion of duplicates, 766 studies were screened by evaluating the title and abstract, focusing on application of piezosurgery in tooth extractions. In total, 131 articles did not meet the inclusion criteria (128 were off-topic; 3 were reviews), leading to 7 articles being selected for qualitative analysis. The selection process and the summary of selected articles are shown in Figure 1 and Table 2, respectively.

### 3.2. Quality Assessment and Risk of Bias in the Included Articles

The risk of bias in the included studies is reported in Table 3. Regarding the bias due to confounding, most studies have a high risk. The bias arising from measurement is a parameter with a low risk of bias. Most studies have a low risk of bias due to bias in the selection of participants. Bias due to post-exposure cannot be calculated due to the high heterogeneity. The bias due to missing data is low in many studies. The bias arising from the measurement of the outcome is low. The bias in the selection of the reported results is high in the majority of studies. The final results show that four studies have a low risk of bias and three have some concerns regarding risk of bias.

## 4. Discussion

In terms of alternatives for traditional rotational devices, piezosurgery has emerged as a less invasive option for extracting mandibular third molars [96,97,98,99,100,101]. Piezosurgery’s ultrasonic vibrations minimize injury to soft tissues while enabling the precise cutting of mineralized tissue [102,103,104,105,106]. Nine clinical investigations comparing piezosurgery with traditional rotary instruments are reviewed in this discussion, with particular attention paid to surgical time, postoperative outcomes (pain, edema, trismus), and bone healing.

### 4.1. Surgical Time

One of the most recurrent conclusions from all of the research is that piezosurgery necessitates far longer operating times than traditional rotary devices [107,108,109,110,111]. According to L. de Freitas Silva et al., the average duration of piezosurgery surgeries was 28.5 ± 3.57 min, while rotary instrument surgeries lasted 17.6 ± 2.95 min. Similar findings were made by H. Arakji et al., who pointed out that piezosurgery takes longer to carry out because of its slower cutting speed, yet its accuracy and lower amount of damage caused to soft tissue may make this longer time acceptable [89,91].

### 4.2. Postoperative Pain and Recovery

There are definite benefits to piezosurgery when it comes to reducing postoperative pain [112,113,114,115,116]. According to J. Rajan et al., patients who had piezosurgery reported much less discomfort, especially in the first three days after the procedure. In addition, patients treated with piezosurgery needed less analgesics than those treated with rotational devices. H. Arakji et al. verified these results, noting on postoperative days 1, 3, and 7 that the VAS pain scores for the piezosurgery group were 3.60, 1.10, and 0.10, respectively, while the rotary group’s levels were 6.70, 3.30, and 1.00. Comparably, in several investigations, E. Mantovani et al. and A. Caputo et al. noted lower pain levels in the piezosurgery group.

### 4.3. Trismus and Mouth Opening

Piezosurgery also helps with reduced mouth opening, or postoperative trismus [117,118,119,120,121]. H. Arakji et al. observed that piezosurgery patients healed faster from trismus, with a mean reduction in the mouth opening of 5.0 mm, compared to 9.7 mm in the rotary group. Better postoperative results were observed when piezosurgery and dexamethasone were combined, according to W. Nehme et al. [92,122].

### 4.4. Swelling and Edema

Swelling reduction is one of the notable benefits of piezosurgery [123,124,125,126,127]. A. Caputo et al., in a meta-analysis, concluded that piezosurgery significantly reduced swelling compared to conventional rotary techniques. E. Mantovani et al. and A. Demirci et al. further reported that the combination of piezosurgery and dexamethasone yielded even better results in reducing postoperative edema, particularly on days 1, 3, and 7 [93,94,95,128].

### 4.5. Bone Healing and Density

Regarding piezosurgery-assisted bone healing, conflicting outcomes have been reported [129,130,131,132,133]. At the four-month point, L. de Freitas Silva et al. and J. Rajan et al. found no discernible variations in bone density between piezosurgery and rotary procedures. On the other hand, the piezosurgery group showed faster bone regeneration than the rotary group, with a mean bone density of 84.45 ± 4.73 at six months postoperatively, as reported by W. Nehm et al. and A. Demirci et al. These results were corroborated by A. Demirci et al., who found that piezosurgery reduces bone loss and promotes bone regeneration.

### 4.6. Comparison Between Piezosurgery and Conventional Rotary Instruments

When piezosurgery and traditional rotating devices are compared, each technique’s advantages and disadvantages are highlighted [134,135,136,137,138,139]. L. de Freitas Silva et al. emphasized that although piezosurgery requires more time, it provides more accuracy and lessens tissue damage in the surrounding area. Because of its accuracy, piezosurgery is particularly well-suited for delicate surgeries such as third molar extractions, where it is essential for preserving soft tissue [140,141,142,143,144,145]. However, the slower cutting speed of piezoelectric devices prolongs surgery time, a drawback noted across several studies, including those by H. Arakji et al. and J. Rajan et al., who both confirmed that rotary instruments were significantly faster in completing extractions [90,91,146,147,148].

Despite the longer operative time, piezosurgery’s less traumatic approach leads to reduced postoperative pain, swelling, and trismus, as reported by E. Mantovani et al. and A. Caputo et al. These advantages make piezosurgery a preferable choice in procedures where patient comfort and postoperative recovery are prioritized over the speed of the surgery [149,150,151,152,153]. Rotary instruments, while faster, may result in more extensive tissue trauma and slower recovery [154,155,156,157,158].

### 4.7. Bone Regeneration

Many studies are focusing on piezosurgery’s capacity to stimulate bone regeneration [159]. Piezosurgery can speed up bone healing and regeneration because it increases osteoblast activity and accelerates cellular metabolism, according to research by W. Nehm et al. and A. Demirci et al. These results were corroborated by A. Demirci et al., who observed increased bone density in patients receiving piezosurgery, especially six months after surgery, when bone density in the piezosurgery group was 84.45 ± 4.73, while it was 74.87 ± 4.03 in the rotary group. Further encouraging faster bone regeneration is the fact that piezosurgery causes less heat injury to the bone than rotational devices, as highlighted by Rahnama et al. and other studies [151,160,161,162,163].

Nevertheless, at the four-month mark, certain investigations, including those by L. de Freitas Silva et al. and J. Rajan et al., reported no appreciable changes in bone density between piezosurgery and rotational procedures [89,90,164]. These differences could result from differences in follow-up times, study designs, and bone healing assessment techniques [165,166,167,168,169]. More thorough trials with longer follow-up periods are required to corroborate the majority of evidence that suggests piezosurgery is superior in encouraging bone regeneration [170,171,172,173,174].

## 5. Limitations and Future Perspectives

The primary constraint of this study is its relatively small sample size, which may limit the generalizability of the findings to broader clinical settings. Additionally, there was no long-term follow-up, reducing the ability to assess the full impact of piezosurgery on bone healing over extended periods. Furthermore, variations in the skill level of clinicians using piezoelectric devices, as well as the learning curve associated with this technology, may have influenced the outcomes. The absence of a standardized protocol for equipment use and clinical indications also presents a limitation, as different setups and surgical techniques may yield variable results. Future studies should consider larger, more diverse populations and longer follow-up periods to validate these findings.

Future studies should focus on enhancing the efficacy of piezosurgery, particularly in reducing surgical time without sacrificing the therapeutic advantages of the procedure. This could be achieved through the optimization of surgical techniques, the development of new, more efficient piezoelectric instruments, and the standardization of operative protocols. Furthermore, the need for additional randomized controlled studies has been emphasized by research, including that of W. Nehme, to confirm the long-term benefits of piezosurgery, especially in relation to bone repair. These studies are crucial for providing robust data that support the use of piezosurgery and for guiding evidence-based clinical decisions.

## 6. Conclusions

In conclusion, this study highlights the clinical relevance of piezosurgery in third molar extractions, particularly its potential to improve postoperative outcomes, such as pain, swelling, and trismus, compared to conventional rotary surgery. The results of this study indicate that patients who have piezosurgery may experience an improvement in their quality of life. Piezosurgery’s longer operating periods, however, provide a practical drawback that may prevent it from being widely used in routine clinical practice. Even with these encouraging outcomes, there are still gaps in the literature. Future studies should concentrate on long-term results in order to more accurately evaluate the overall advantages of piezosurgery. Larger-scale research is also required to validate these results and investigate different patient responses to various surgical approaches, especially in more intricate extractions. Clinicians can have a better understanding of piezosurgery’s potential and its role in standard dental treatment by focusing on these topics.

## Figures and Tables

**Table 1:** Database search indicators.

Article screening strategy	Keywords: A: piezosurgery; B: tooth extraction
	Boolean Indicators: A and B
	Timespan: 1 January 2004 to 28 August 2024
	Electronic databases: Pubmed; Scopus; Web of Science

**Table 2:** Descriptive summary of article selection.

First Author (Year)	Type of Study	Aim of the Study	Materials and Methods	Results
L. de Freitas Silva et al. (2019) [89]	Randomized Controlled Trial (Split-mouth)	To compare the effectiveness of piezosurgery vs. conventional techniques in third molar extractions	Split-mouth study with 15 patients (18–30 years). One side treated with piezosurgery, the other with conventional methods. Bone density and healing assessed via radiographs.	There was no significant difference in bone density between the two techniques.
J. Rajan et al. (2009) [90]	Randomized Controlled Trial	To compare the efficiency of piezosurgery and conventional rotary instruments in third molar extraction	20 patients (aged >18) randomized to piezosurgery or rotary instruments. Pain, trismus, and swelling were assessed on days 1, 3, 7, and 14.	Piezosurgery led to less pain, swelling, and faster recovery but took longer. The rotary group had higher analgesic use and soft tissue damage.
H. Arakji et al.(2016) [91]	Randomized Controlled Trial (Split-mouth)	To compare the clinical and radiographical outcomes of piezosurgery and conventional rotary instruments for impacted third molar extractions	20 male patients (split-mouth). Pain, trismus, swelling, and bone density measured using VAS, calipers, and radiographs.	Piezosurgery took longer but showed less pain, swelling, and better bone density (*p* ≤ 0.0001)
W. Nehme et al. (2021) [92]	Randomized Controlled Clinical Trial	To evaluate the effects of piezosurgery and dexamethasone on postoperative outcomes after impacted mandibular third molar surgery	80 patients (15–30 years) divided into four groups: piezosurgery or conventional surgery with/without dexamethasone. Pain and trismus were assessed using VAS and calipers	Piezosurgery with dexamethasone provided the best outcomes, with less pain and trismus on days 1 and 3.
A. Demirci et al. (2022) [93]	Randomized Controlled Clinical Trial	To compare the effectiveness of piezosurgery vs. conventional rotary surgery for impacted mandibular third molar extractions.	20 patients (40 teeth, 18–35 years). Piezosurgery vs. rotary surgery. Pain, trismus, swelling, and operative time measured. Quality of life assessed via OHIP-14.	Piezosurgery had less pain, swelling, and trismus by days 1 and 3, but operative time was shorter with rotary instruments
A. Caputo et al. (2023) [94]	Randomized, Split-Mouth, Single-Blind Study	To evaluate the postoperative facial swelling after lower third molar extraction using piezoelectric surgery vs. rotary instruments.	22 patients (18–40 years). Symmetrical lower third molar extractions using piezosurgery vs. rotary. Swelling assessed with 3D scans at days 3 and 7.	No significant difference in swelling between methods. No adverse reactions reported.
E. Mantovani et al. (2014) [95]	Single-center, randomized, split-mouth study	To compare the efficacy and postoperative outcomes of piezosurgery vs. conventional bur techniques for mandibular third molar removal.	100 patients underwent bilateral mandibular molar extractions using piezosurgery or bur techniques. Pain, swelling, and surgery duration were measured.	Piezosurgery resulted in less pain and swelling but longer surgery time. It was preferred by 65% of patients.

**Table 3:** Bias assessment.

Authors	D1	D2	D3	D4	D5	D6	D7	Overall
L. de Freitas Silva et al. (2019) [89]	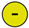	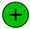	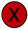	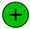	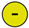	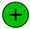	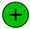	
J. Rajan et al. (2009) [90]	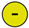	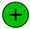	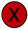	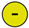	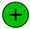	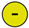	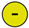	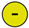
H. Arakji et al.(2016) [91]	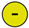	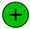	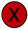	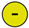	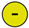	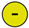	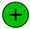	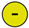
W. Nehme et al. (2021) [92]	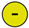	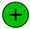	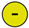	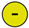	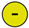	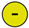	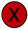	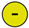
A. Demirci et al. (2022) [93]	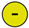	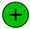	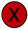	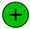	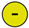	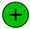	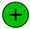	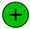
A. Caputo et al. (2023) [94]	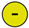	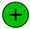	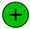	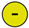	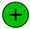	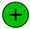	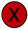	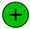
E. Mantovani et al. (2014) [95]	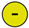	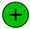	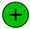	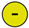	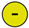	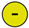	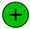	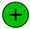

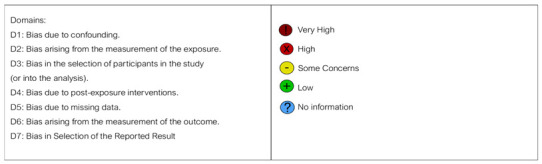
Domains: D1: Bias due to confounding. D2: Bias arising from the measurement of the exposure. D3: Bias in the selection of participants in the study (or into the analysis). D4: Bias due to post-exposure interventions. D5: Bias due to missing data. D6: Bias arising from the measurement of the outcome. D7: Bias in Selection of the Reported Result. 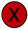 High 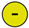 Some Concerns 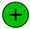 Low.

## Data Availability

Not applicable.

## Abbreviations

CBCT	Cone-beam computed tomography
OPG	Orthopantomogram
PRISMA	Preferred Reporting Items for Systematic Reviews and Meta-Analyses
PROSPERO	The International Prospective Register of Systematic Reviews
RCT	Randomized controlled trial
VAS	Visual analog scale

## References

[1] Genç B.G.Ç., Orhan K., Or S. (2023). A Clinical Comparison of Er:YAG Laser, Piezosurgery, and Conventional Bur Methods in the Impacted Third Molar Surgery. Photobiomodulation Photomed. Laser Surg..

[2] Erdem M.K., Cambazoglu M. (2024). A Comparative Analysis of Postoperative Morbidity and Alveolar Bone Regeneration Following Surgical Extraction of Impacted Lower Third Molar Teeth Using Piezosurgery and Conventional Instruments: A Split-Mouth Clinical Investigation. Eur. J. Med. Res..

[3] Gulnahar Y., Alpan A.-L. (2021). Comparison of Postoperative Morbidity between Piezoelectric Surgery and Conventional Rotary Instruments in Mandibular Third Molar Surgery: A Split-Mouth Clinical Study. Med. Oral Patol. Oral Cir. Bucal.

[4] Young L., Brown T., Lamont T.J. (2020). A Comparison of Techniques for the Explantation of Osseointegrated Dental Implants. Evid. Based Dent..

[5] Saraiva Amaral J., Marto C.M., Farias J., Alves Pereira D., Ermida J., Banaco Á., Campos Felino A., Caramelo F., Matos S. (2022). A Pilot Randomized Controlled Clinical Trial Comparing Piezo Versus Conventional Rotary Surgery for Removal of Impacted Mandibular Third Molars. Bioengineering.

[6] Aksakalli S., Calik B., Kara B., Ezirganli S. (2016). Accelerated Tooth Movement with Piezocision and Its Periodontal-Transversal Effects in Patients with Class II Malocclusion. Angle Orthod..

[7] Seymour R.A., Charlton J.E., Phillips M.E. (1983). An Evaluation of Dental Pain Using Visual Analogue Scales and the Mcgill Pain Questionnaire. J. Oral Maxillofac. Surg..

[8] Menziletoglu D., Basturk F., Isik B.K., Esen A. (2020). A Prospective Split-Mouth Clinical Study: Comparison of Piezosurgery and Conventional Rotary Instruments in Impacted Third Molar Surgery. Oral Maxillofac. Surg..

[9] Osborn T.P., Frederickson G., Small I.A., Torgerson T.S. (1985). A Prospective Study of Complications Related to Mandibular Third Molar Surgery. J. Oral Maxillofac. Surg..

[10] Barone A., Marconcini S., Giacomelli L., Rispoli L., Calvo J.L., Covani U. (2010). A Randomized Clinical Evaluation of Ultrasound Bone Surgery versus Traditional Rotary Instruments in Lower Third Molar Extraction. J. Oral Maxillofac. Surg..

[11] Inchingolo F., Inchingolo A.D., Palumbo I., Guglielmo M., Balestriere L., Casamassima L., Ciccarese D., Marotti P., Mancini A., Palermo A. (2024). Management of Physiological Gingival Melanosis by Diode Laser Depigmentation versus Surgical Scalpel: A Systematic Review. Dent. Rev..

[12] Malcangi G., Patano A., Morolla R., De Santis M., Piras F., Settanni V., Mancini A., Di Venere D., Inchingolo F., Inchingolo A.D. (2023). Analysis of Dental Enamel Remineralization: A Systematic Review of Technique Comparisons. Bioengineering.

[13] McGovern J.A., Griffin M., Hutmacher D.W. (2018). Animal Models for Bone Tissue Engineering and Modelling Disease. Dis. Models Mech..

[14] Schafrum Macedo A., Cezaretti Feitosa C., Yoiti Kitamura Kawamoto F., Vinicius Tertuliano Marinho P., Dos Santos Dal-Bó Í., Fiuza Monteiro B., Prado L., Bregadioli T., Antonio Covino Diamante G., Ricardo Auada Ferrigno C. (2019). Animal Modeling in Bone Research-Should We Follow the White Rabbit?. Anim. Models Exp. Med..

[15] Wancket L.M. (2015). Animal Models for Evaluation of Bone Implants and Devices: Comparative Bone Structure and Common Model Uses. Vet. Pathol..

[16] Inchingolo A.D., Inchingolo A.M., Malcangi G., Avantario P., Azzollini D., Buongiorno S., Viapiano F., Campanelli M., Ciocia A.M., De Leonardis N. (2022). Effects of Resveratrol, Curcumin and Quercetin Supplementation on Bone Metabolism-A Systematic Review. Nutrients.

[17] Malcangi G., Patano A., Ciocia A.M., Netti A., Viapiano F., Palumbo I., Trilli I., Guglielmo M., Inchingolo A.D., Dipalma G. (2023). Benefits of Natural Antioxidants on Oral Health. Antioxidants.

[18] Inchingolo A.M., Malcangi G., Costa S., Fatone M.C., Avantario P., Campanelli M., Piras F., Patano A., Ferrara I., Di Pede C. (2023). Tooth Complications after Orthodontic Miniscrews Insertion. Int. J. Environ. Res. Public Health.

[19] Monaco G., Staffolani C., Gatto M.R., Checchi L. (1999). Antibiotic Therapy in Impacted Third Molar Surgery. Eur. J. Oral Sci..

[20] Gargiulo Isacco C., Inchingolo A.D., Nguyen Cao K.D., Malcangi G., Paduanelli G., Pham Hung V., Tran Cong T., Bordea I.R., Scarano A., Laforgia A. (2021). The Bad Relationship, Osteo-Decay and Diabetes Type 2 Searching for a Link: A Literature Review. J. Biol. Regul. Homeost. Agents.

[21] Inchingolo F., Dipalma G., Paduanelli G., De Oliveira L.A., Inchingolo A.M., Georgakopoulos P.I., Inchingolo A.D., Malcangi G., Athanasiou E., Fotopoulou E. (2019). Computer-Based Quantification of an Atraumatic Sinus Augmentation Technique Using CBCT. J. Biol. Regul. Homeost. Agents.

[22] Inchingolo F., Tatullo M., Abenavoli F.M., Marrelli M., Inchingolo A.D., Corelli R., Inchingolo A.M., Dipalma G. (2010). Eyelid Bags. Head Face Med..

[23] Inchingolo F., Santacroce L., Ballini A., Topi S., Dipalma G., Haxhirexha K., Bottalico L., Charitos I.A. (2020). Oral Cancer: A Historical Review. Int. J. Environ. Res. Public Health.

[24] Li D., Guo C.B., Liu Y., Wang E.B. (2016). Applicational evaluation of split tooth extractions of upper molars using piezosurgery. Beijing Da Xue Xue Bao Yi Xue Ban.

[25] Dipalma G., Inchingolo A.D., Inchingolo A.M., Piras F., Carpentiere V., Garofoli G., Azzollini D., Campanelli M., Paduanelli G., Palermo A. (2023). Artificial Intelligence and Its Clinical Applications in Orthodontics: A Systematic Review. Diagnostics.

[26] Ruta D.A., Bissias E., Ogston S., Ogden G.R. (2000). Assessing Health Outcomes after Extraction of Third Molars: The Postoperative Symptom Severity (PoSSe) Scale. Br. J. Oral Maxillofac. Surg..

[27] Malcangi G., Patano A., Guglielmo M., Sardano R., Palmieri G., Di Pede C., de Ruvo E., Inchingolo A.D., Mancini A., Inchingolo F. (2023). Precision Medicine in Oral Health and Diseases: A Systematic Review. J. Pers. Med..

[28] Inchingolo A.M., Patano A., Piras F., de Ruvo E., Ferrante L., Noia A.D., Dongiovanni L., Palermo A., Inchingolo F., Inchingolo A.D. (2023). Orthognathic Surgery and Relapse: A Systematic Review. Bioengineering.

[29] Inchingolo A.M., Inchingolo A.D., Carpentiere V., Del Vecchio G., Ferrante L., Di Noia A., Palermo A., Di Venere D., Dipalma G., Inchingolo F. (2023). Predictability of Dental Distalization with Clear Aligners: A Systematic Review. Bioengineering.

[30] Grossi G.B., Maiorana C., Garramone R.A., Borgonovo A., Creminelli L., Santoro F. (2007). Assessing Postoperative Discomfort After Third Molar Surgery: A Prospective Study. J. Oral Maxillofac. Surg..

[31] Giraud J.Y., Villemin S., Darmana R., Cahuzac J.P., Autefage A., Morucci J.P. (1991). Bone Cutting. Clin. Phys. Physiol. Meas..

[32] Inchingolo A.M., Patano A., Piras F., Mancini A., Inchingolo A.D., Paduanelli G., Inchingolo F., Palermo A., Dipalma G., Malcangi G. (2023). Interconnection between Microbiota–Gut–Brain Axis and Autism Spectrum Disorder Comparing Therapeutic Options: A Scoping Review. Microorganisms.

[33] Romeo U., Del Vecchio A., Palaia G., Tenore G., Visca P., Maggiore C. (2009). Bone Damage Induced by Different Cutting Instruments--an in Vitro Study. Braz. Dent. J..

[34] Li Y., Chen S.-K., Li L., Qin L., Wang X.-L., Lai Y.-X. (2015). Bone Defect Animal Models for Testing Efficacy of Bone Substitute Biomaterials. J. Orthop. Transl..

[35] Inchingolo A.M., Patano A., De Santis M., Del Vecchio G., Ferrante L., Morolla R., Pezzolla C., Sardano R., Dongiovanni L., Inchingolo F. (2023). Comparison of Different Types of Palatal Expanders: Scoping Review. Children.

[36] Inchingolo A.M., Malcangi G., Ferrante L., Del Vecchio G., Viapiano F., Inchingolo A.D., Mancini A., Annicchiarico C., Inchingolo F., Dipalma G. (2023). Surface Coatings of Dental Implants: A Review. J. Funct. Biomater..

[37] Akçay H., Tatar B., Kuru K., Gözlüklü Ö., Ulu M. (2019). Bone Flap Technique With Piezosurgery for Impacted Teeth Extraction and Bone Cysts Removal Without Additional Fixation. J. Craniofac. Surg..

[38] Anesi A., Di Bartolomeo M., Pellacani A., Ferretti M., Cavani F., Salvatori R., Nocini R., Palumbo C., Chiarini L. (2020). Bone Healing Evaluation Following Different Osteotomic Techniques in Animal Models: A Suitable Method for Clinical Insights. Appl. Sci..

[39] Lajolo C., Valente N.A., Romandini W.G., Petruzzi M., Verdugo F., D’Addona A. (2018). Bone Heat Generated Using Conventional Implant Drills versus Piezosurgery Unit during Apical Cortical Plate Perforation. J. Periodontol..

[40] Vucetic M., Roganovic J., Freilich M., Shafer D., Milic M., DJukic L., Petrovic N., Markovic E., Markovic A., Brkovic B. (2021). Bone microRNA-21 as Surgical Stress Parameter Is Associated with Third Molar Postoperative Discomfort. Clin. Oral Investig..

[41] Inchingolo F., Inchingolo A.M., Piras F., Ferrante L., Mancini A., Palermo A., Inchingolo A.D., Dipalma G. (2024). The Interaction between Gut Microbiome and Bone Health. Curr. Opin. Endocrinol. Diabetes Obes..

[42] Inchingolo F., Inchingolo A.D., Palumbo I., Trilli I., Guglielmo M., Mancini A., Palermo A., Inchingolo A.M., Dipalma G. (2024). The Impact of Cesarean Section Delivery on Intestinal Microbiota: Mechanisms, Consequences, and Perspectives—A Systematic Review. Int. J. Mol. Sci..

[43] Bellucci D., Cannillo V., Anesi A., Salvatori R., Chiarini L., Manfredini T., Zaffe D. (2018). Bone Regeneration by Novel Bioactive Glasses Containing Strontium and/or Magnesium: A Preliminary In-Vivo Study. Materials.

[44] Schindeler A., McDonald M.M., Bokko P., Little D.G. (2008). Bone Remodeling during Fracture Repair: The Cellular Picture. Semin. Cell Dev. Biol..

[45] Scolozzi P. (2022). Buccal Corticotomy Using Piezosurgery as a Surgical Approach for Removal of Deeply Impacted Mandibular Teeth: An Alternative Procedure to Avoid Pitfalls Associated with the Conventional Technique. J. Stomatol. Oral Maxillofac. Surg..

[46] Inchingolo A.D., Dipalma G., Viapiano F., Netti A., Ferrara I., Ciocia A.M., Mancini A., Di Venere D., Palermo A., Inchingolo A.M. (2024). Celiac Disease-Related Enamel Defects: A Systematic Review. J. Clin. Med..

[47] Sharma A.K., Gupta A., Pabari H.P., Pathak S.K., Odedra N.H., Beniwal J., Arora K.S. (2023). Comparative and Clinical Evaluation between Piezoelectric and Conventional Rotary Techniques for Mandibular Impacted Third Molar Extraction. Natl. J. Maxillofac. Surg..

[48] Goyal M., Marya K., Jhamb A., Chawla S., Sonoo P.R., Singh V., Aggarwal A. (2012). Comparative Evaluation of Surgical Outcome after Removal of Impacted Mandibular Third Molars Using a Piezotome or a Conventional Handpiece: A Prospective Study. Br. J. Oral Maxillofac. Surg..

[49] Guo Z., Zhang H., Li Y., Li X., Liu Y., Wang Y., Yuan C., Liu X. (2012). Comparative study of complications among routine method, high speed turbine handpiece and piezosurgery device after extraction of impacted wisdom teeth. Shanghai Kou Qiang Yi Xue.

[50] Bennardo F., Barone S., Vocaturo C., Gheorghe D.N., Cosentini G., Antonelli A., Giudice A. (2023). Comparison between Magneto-Dynamic, Piezoelectric, and Conventional Surgery for Dental Extractions: A Pilot Study. Dent. J..

[51] Chang H.-H., Lee M.-S., Hsu Y.-C., Tsai S.-J., Lin C.-P. (2015). Comparison of Clinical Parameters and Environmental Noise Levels between Regular Surgery and Piezosurgery for Extraction of Impacted Third Molars. J. Formos. Med. Assoc..

[52] Sagheb K., Kumar V.V., Azaripour A., Walter C., Al-Nawas B., Kämmerer P.W. (2017). Comparison of Conventional Twist Drill Protocol and Piezosurgery for Implant Insertion: An Ex Vivo Study on Different Bone Types. Clin. Oral Implant. Res..

[53] Gabrić D., Blašković M., Gjorgijevska E., Mladenov M., Tašič B., Jurič I.B., Ban T. (2016). Evaluation of Bone Healing After Osteotomies Prepared With Er:YAG Laser in Contact and Noncontact Modes and Piezosurgery—An Animal Study. J. Oral Maxillofac. Surg..

[54] Bulloch S.E., Olsen R.G., Bulloch B. (2012). Comparison of Heat Generation between Internally Guided (Cannulated) Single Drill and Traditional Sequential Drilling with and without a Drill Guide for Dental Implants. Int. J. Oral Maxillofac. Implant..

[55] Mehmanparast H., Petit Y., Mac-Thiong J.-M. (2015). Comparison of Pedicle Screw Loosening Mechanisms and the Effect on Fixation Strength. J. Biomech. Eng..

[56] Baqain Z.H., Karaky A.A., Sawair F., Khraisat A., Duaibis R., Rajab L.D. (2008). Frequency Estimates and Risk Factors for Postoperative Morbidity after Third Molar Removal: A Prospective Cohort Study. J. Oral Maxillofac. Surg..

[57] Rashid N., Subbiah V., Agarwal P., Kumar S., Bansal A., Neeraj, Reddy S.G., Chug A. (2020). Comparison of Piezosurgery and Conventional Rotatory Technique in Transalveolar Extraction of Mandibular Third Molars: A Pilot Study. J. Oral Biol. Craniofac. Res..

[58] Shetty L., Gangwani K., Londhe U., Bharadwaj S., Bakri M.M.H., Alamoudi A., Reda R., Bhandi S., Raj A.T., Patil S. (2022). Comparison of the C-Reactive Protein Level and Visual Analog Scale Scores between Piezosurgery and Rotatory Osteotomy in Mandibular Impacted Third Molar Extraction. Life.

[59] Mcfall T.A., Yamane G.M., Burnett G.W. (1961). Comparison of the Cutting Effect on Bone of an Ultrasonic Cutting Device and Rotary Burs. J. Oral Surg. Anesth. Hosp. Dent. Serv..

[60] UStün Y., Erdogan O., Esen E., Karsli E.D. (2003). Comparison of the Effects of 2 Doses of Methylprednisolone on Pain, Swelling, and Trismus after Third Molar Surgery. Oral Surg. Oral Med. Oral Pathol. Oral Radiol. Endod..

[61] Jiang S., Zhou B., Li Z., Gao J., Wang P. (2023). Comparison of the Effects of Two Extraction Methods on the Alveolar Ridge Preservation of Maxillary Anterior Teeth. Pak. J. Med. Sci..

[62] Sisk A.L., Hammer W.B., Shelton D.W., Joy E.D. (1986). Complications Following Removal of Impacted Third Molars: The Role of the Experience of the Surgeon. J. Oral Maxillofac. Surg..

[63] Blagova B., Krastev D., Malinova L. (2023). Conventional Drilling versus Ultrasound and Laser Osteotomy in Mandibular Third Molar Surgery: A Comparative Study. Lasers Surg. Med..

[64] Markiewicz M.R., Brady M.F., Ding E.L., Dodson T.B. (2008). Corticosteroids Reduce Postoperative Morbidity after Third Molar Surgery: A Systematic Review and Meta-Analysis. J. Oral Maxillofac. Surg..

[65] Landes C.A., Stübinger S., Rieger J., Williger B., Ha T.K.L., Sader R. (2008). Critical Evaluation of Piezoelectric Osteotomy in Orthognathic Surgery: Operative Technique, Blood Loss, Time Requirement, Nerve and Vessel Integrity. J. Oral Maxillofac. Surg..

[66] Troedhan A., Mahmoud Z.T., Wainwright M., Khamis M.M., Troedhan A., Mahmoud Z.T., Wainwright M., Khamis M.M. (2017). Cutting Bone with Drills, Burs, Lasers and Piezotomes: A Comprehensive Systematic Review and Recommendations for the Clinician. Int. J. Oral Craniofacial Sci..

[67] Malavasi G., Salvatori R., Zambon A., Lusvardi G., Rigamonti L., Chiarini L., Anesi A. (2019). Cytocompatibility of Potential Bioactive Cerium-Doped Glasses Based on 45S5. Materials.

[68] Preti G., Martinasso G., Peirone B., Navone R., Manzella C., Muzio G., Russo C., Canuto R.A., Schierano G. (2007). Cytokines and Growth Factors Involved in the Osseointegration of Oral Titanium Implants Positioned Using Piezoelectric Bone Surgery versus a Drill Technique: A Pilot Study in Minipigs. J. Periodontol..

[69] Ferretti M., Palumbo C., Contri M., Marotti G. (2002). Static and Dynamic Osteogenesis: Two Different Types of Bone Formation. Anat. Embryol..

[70] Al-Moraissi E.A., Elmansi Y.A., Al-Sharaee Y.A., Alrmali A.E., Alkhutari A.S. (2016). Does the Piezoelectric Surgical Technique Produce Fewer Postoperative Sequelae after Lower Third Molar Surgery than Conventional Rotary Instruments? A Systematic Review and Meta Analysis. Int. J. Oral Maxillofac. Surg..

[71] Pandey R.K., Panda S.S. (2013). Drilling of Bone: A Comprehensive Review. J. Clin. Orthop. Trauma.

[72] Esteves J.C., Marcantonio E., de Souza Faloni A.P., Rocha F.R.G., Marcantonio R.A., Wilk K., Intini G. (2013). Dynamics of Bone Healing after Osteotomy with Piezosurgery or Conventional Drilling—Histomorphometrical, Immunohistochemical, and Molecular Analysis. J. Transl. Med..

[73] O’Donnell R.J., Deutsch T.F., Flotte R.J., Lorente C.A., Tomford W.W., Mankin H.J., Schomacker K.T. (1996). Effect of Er:YAG Laser Holes on Osteoinduction in Demineralized Rat Calvarial Allografts. J. Orthop Res..

[74] Forouzanfar T., Sabelis A., Ausems S., Baart J.A., van der Waal I. (2008). Effect of Ice Compression on Pain after Mandibular Third Molar Surgery: A Single-Blind, Randomized Controlled Trial. Int. J. Oral Maxillofac. Surg..

[75] Bullon B., Bueno E.F., Herrero M., Fernandez-Palacin A., Rios J.V., Bullon P., Gil F.J. (2015). Effect of Irrigation and Stainless Steel Drills on Dental Implant Bed Heat Generation. J. Mater. Sci. Mater. Med..

[76] Kim D.H., Kang H., Jin H.J., Hwang S.H. (2019). Effect of Piezoelectric Osteotomy on Postoperative Oedema and Ecchymosis after Rhinoplasty. Clin. Otolaryngol..

[77] Grossi G.B., Maiorana C., Garramone R.A., Borgonovo A., Beretta M., Farronato D., Santoro F. (2007). Effect of Submucosal Injection of Dexamethasone on Postoperative Discomfort After Third Molar Surgery: A Prospective Study. J. Oral Maxillofac. Surg..

[78] Al-Khateeb T.H., Nusair Y. (2008). Effect of the Proteolytic Enzyme Serrapeptase on Swelling, Pain and Trismus after Surgical Extraction of Mandibular Third Molars. Int. J. Oral Maxillofac. Surg..

[79] Pavone C., Scardueli C.R., de Oliveira G.J.P.L., Cerri P.S., Marcantonio Junior E., Marcantonio R.A.C. (2019). Effects of an Er, Cr:YSGG Laser on Bone Regeneration in Critical-Sized Calvarial Defects of Rats Exposed to Inhalation of Cigarette Smoke. Photobiomodul. Photomed. Laser Surg..

[80] Brasseur M., Brogniez V., Grégoire V., Reychler H., Lengelé B., D’Hoore W., Nyssen-Behets C. (2006). Effects of Irradiation on Bone Remodelling around Mandibular Implants: An Experimental Study in Dogs. Int. J. Oral Maxillofac. Surg..

[81] Uyanık L.O., Bilginaylar K., Etikan İ. (2015). Effects of Platelet-Rich Fibrin and Piezosurgery on Impacted Mandibular Third Molar Surgery Outcomes. Head Face Med..

[82] Yang B.-E., Girod S. (2014). Efficacy of Bone Healing in Calvarial Defects Using Piezoelectric Surgical Instruments. J. Craniofacial Surg..

[83] Tunçer N.I., Arman-Özçirpici A., Oduncuoglu B.F., Göçmen J.S., Kantarci A. (2017). Efficiency of Piezosurgery Technique in Miniscrew Supported En-Masse Retraction: A Single-Centre, Randomized Controlled Trial. Eur. J. Orthod..

[84] Agarwal E., Masamatti S.S., Kumar A. (2014). Escalating Role of Piezosurgery in Dental Therapeutics. J. Clin. Diagn. Res..

[85] Gabrić Pandurić D., Bago I., Katanec D., Zabkar J., Miletić I., Anić I. (2012). Comparison of Er:YAG Laser and Surgical Drill for Osteotomy in Oral Surgery: An Experimental Study. J. Oral Maxillofac. Surg..

[86] Abbas N.H., Sabet N.E., Hassan I.T. (2016). Evaluation of Corticotomy-Facilitated Orthodontics and Piezocision in Rapid Canine Retraction. Am. J. Orthod. Dentofac. Orthop..

[87] Neupert E.A., Lee J.W., Philput C.B., Gordon J.R. (1992). Evaluation of Dexamethasone for Reduction of Postsurgical Sequelae of Third Molar Removal. J. Oral Maxillofac. Surg..

[88] Page M.J., McKenzie J.E., Bossuyt P.M., Boutron I., Hoffmann T.C., Mulrow C.D., Shamseer L., Tetzlaff J.M., Akl E.A., Brennan S.E. (2021). The PRISMA 2020 Statement: An Updated Guideline for Reporting Systematic Reviews. BMJ.

[89] de Freitas Silva L., Ribeiro de Carvalho Reis E.N., Oliveira Souza B.C., Egas L.S., Aranega A.M., Ponzoni D. (2019). Alveolar Repair after the Use of Piezosurgery in the Removal of Lower Third Molars: A Prospective Clinical, Randomised, Double-Blind, Split-Mouth Study. Br. J. Oral Maxillofac. Surg..

[90] Ergina P.L., Cook J.A., Blazeby J.M., Boutron I., Clavien P.-A., Reeves B.C., Seiler C.M., Altman D.G., Aronson J.K., Balliol Collaboration (2009). Challenges in Evaluating Surgical Innovation. Lancet.

[91] Arakji H., Shokry M., Aboelsaad N. (2016). Comparison of Piezosurgery and Conventional Rotary Instruments for Removal of Impacted Mandibular Third Molars: A Randomized Controlled Clinical and Radiographic Trial. Int. J. Dent..

[92] Nehme W., Fares Y., Abou-Abbas L. (2021). Piezo-Surgery Technique and Intramuscular Dexamethasone Injection to Reduce Postoperative Pain after Impacted Mandibular Third Molar Surgery: A Randomized Clinical Trial. BMC Oral Health.

[93] Demirci A., Bayram F., Dergin G. (2024). Piezosurgery versus Conventional Rotary Surgery for Impacted Third Molars: A Randomised, Split-Mouth, Clinical Pilot Trial. Med. Oral Patol. Oral Cir. Bucal.

[94] Caputo A., Rubino E., Marcianò A., Peditto M., Bellocchio A.M., Nucera R., Oteri G. (2023). Three-Dimensional Facial Swelling Evaluation of Piezo-Electric vs Conventional Drilling Bur Surgery of Impacted Lower Third Molar: A Randomized Clinical Trial. BMC Oral Health.

[95] Mantovani E., Arduino P.G., Schierano G., Ferrero L., Gallesio G., Mozzati M., Russo A., Scully C., Carossa S. (2014). A Split-Mouth Randomized Clinical Trial to Evaluate the Performance of Piezosurgery Compared with Traditional Technique in Lower Wisdom Tooth Removal. J. Oral Maxillofac. Surg..

[96] Bilginaylar K., Uyanik L.O. (2016). Evaluation of the Effects of Platelet-Rich Fibrin and Piezosurgery on Outcomes after Removal of Impacted Mandibular Third Molars. Br. J. Oral Maxillofac. Surg..

[97] Ge J., Yang C., Zheng J.-W., He D.-M., Zheng L.-Y., Hu Y.-K. (2014). Four Osteotomy Methods with Piezosurgery to Remove Complicated Mandibular Third Molars: A Retrospective Study. J. Oral Maxillofac. Surg..

[98] Pathak S., Vashisth S., Mishra S., Singh S.P., Sharma S. (2014). Grading of Extraction and Its Relationship with Post-Operative Pain and Trismus, along with Proposed Grading for Trismus. J. Clin. Diagn. Res..

[99] Scott J., Huskisson E.C. (1976). Graphic Representation of Pain. Pain.

[100] Favero V., Sakuma S., Apaza Alccayhuaman K.A., Benedetto G.A., Bengazi F., Botticelli D. (2018). Healing at Sites Prepared Using Different Drilling Protocols. An Experimental Study in the Tibiae of Sheep. PLoS ONE.

[101] Patano A., Malcangi G., Santis M.D., Morolla R., Settanni V., Piras F., Inchingolo A.D., Mancini A., Inchingolo F., Dipalma G. (2023). Conservative Treatment of Dental Non-Carious Cervical Lesions: A Scoping Review. Biomedicines.

[102] Ma L., Stübinger S., Liu X.L., Schneider U.A., Lang N.P. (2013). Healing of Osteotomy Sites Applying Either Piezosurgery or Two Conventional Saw Blades: A Pilot Study in Rabbits. Int. Orthop..

[103] Eriksson A.R., Albrektsson T. (1983). Temperature Threshold Levels for Heat-Induced Bone Tissue Injury: A Vital-Microscopic Study in the Rabbit. J. Prosthet. Dent..

[104] Möhlhenrich S.C., Modabber A., Steiner T., Mitchell D.A., Hölzle F. (2015). Heat Generation and Drill Wear during Dental Implant Site Preparation: Systematic Review. Br. J. Oral Maxillofac. Surg..

[105] Marković A., Mišić T., Miličić B., Calvo-Guirado J.L., Aleksić Z., Ðinić A. (2013). Heat Generation during Implant Placement in Low-Density Bone: Effect of Surgical Technique, Insertion Torque and Implant Macro Design. Clin. Oral Implant. Res..

[106] Cordioli G., Majzoub Z. (1997). Heat Generation during Implant Site Preparation: An in Vitro Study. Int. J. Oral Maxillofac. Implant..

[107] Gabka J., Matsumura T. (1971). Measuring techniques and clinical testing of an anti-inflammatory agent (tantum). Munch. Med. Wochenschr..

[108] Nelson J.S., Orenstein A., Liaw L.H., Berns M.W. (1989). Mid-Infrared Erbium:YAG Laser Ablation of Bone: The Effect of Laser Osteotomy on Bone Healing. Lasers Surg. Med..

[109] Zhang Y., Xu L., Wang C., Chen Z., Han S., Chen B., Chen J. (2019). Mechanical and Thermal Damage in Cortical Bone Drilling In Vivo. J. Eng. Med..

[110] Crovace A.M., Luzzi S., Lacitignola L., Fatone G., Giotta Lucifero A., Vercellotti T., Crovace A. (2020). Minimal Invasive Piezoelectric Osteotomy in Neurosurgery: Technic, Applications, and Clinical Outcomes of a Retrospective Case Series. Vet. Sci..

[111] Iyer S.S., Haribabu P.K. (2013). Minimizing Alveolar Bone Loss during and after Extractions (Part I)—Review of Techniques: Atraumatic Extraction, Root Retention. Alpha Omegan.

[112] Zizzari V.L., Berardi D., Congedi F., Tumedei M., Cataldi A., Perfetti G. (2015). Morphological Aspect and iNOS and Bax Expression Modification in Bone Tissue Around Dental Implants Positioned Using Piezoelectric Bone Surgery Versus Conventional Drill Technique. J. Craniofac. Surg..

[113] Vercellotti T., Podesta A. (2007). Orthodontic Microsurgery: A New Surgically Guided Technique for Dental Movement. Int. J. Periodontics Restor. Dent..

[114] Brånemark P.I. (1983). Osseointegration and Its Experimental Background. J. Prosthet. Dent..

[115] Vercellotti T., Nevins M.L., Kim D.M., Nevins M., Wada K., Schenk R.K., Fiorellini J.P. (2005). Osseous Response Following Resective Therapy with Piezosurgery. Int. J. Periodontics Restor. Dent..

[116] Palumbo C., Ferretti M., Marotti G. (2004). Osteocyte Dendrogenesis in Static and Dynamic Bone Formation: An Ultrastructural Study. Anat. Rec. A Discov. Mol. Cell. Evol. Biol..

[117] Sivolella S., Berengo M., Bressan E., Di Fiore A., Stellini E. (2011). Osteotomy for Lower Third Molar Germectomy: Randomized Prospective Crossover Clinical Study Comparing Piezosurgery and Conventional Rotatory Osteotomy. J. Oral Maxillofac. Surg..

[118] Flanagan D. (2010). Osteotomy Irrigation: Is It Necessary?. Implant Dent..

[119] Morris C.R., Jerman A.C. (1971). Panoramic Radiographic Survey: A Study of Embedded Third Molars. J. Oral Surg..

[120] Luvan M., Kanthan S., Roshan G., Saw A. (2015). Pattern of Cortical Fracture Following Corticotomy for Distraction Osteogenesis. Malays. Orthop. J..

[121] Rood J.P. (1992). Permanent Damage to Inferior Alveolar and Lingual Nerves during the Removal of Impacted Mandibular Third Molars. Comparison of Two Methods of Bone Removal. Br. Dent. J..

[122] Inchingolo A.D., Gargiulo C.I., Malcangi G., Ciocia A.M., Patano A., Azzollini D., Piras F., Barile G., Settanni V., Mancini A. (2022). Diagnosis of SARS-CoV-2 during the Pandemic by Multiplex RT-rPCR hCoV Test: Future Perspectives. Pathogens.

[123] Robiony M., Polini F., Costa F., Vercellotti T., Politi M. (2004). Piezoelectric Bone Cutting in Multipiece Maxillary Osteotomies. J. Oral Maxillofac. Surg..

[124] Cicciù M., Stacchi C., Fiorillo L., Cervino G., Troiano G., Vercellotti T., Herford A.S., Galindo-Moreno P., Di Lenarda R. (2021). Piezoelectric Bone Surgery for Impacted Lower Third Molar Extraction Compared with Conventional Rotary Instruments: A Systematic Review, Meta-Analysis, and Trial Sequential Analysis. Int. J. Oral Maxillofac. Surg..

[125] Schaller B.J., Gruber R., Merten H.A., Kruschat T., Schliephake H., Buchfelder M., Ludwig H.C. (2005). Piezoelectric Bone Surgery: A Revolutionary Technique for Minimally Invasive Surgery in Cranial Base and Spinal Surgery? Technical Note. Neurosurgery.

[126] Badenoch-Jones E.K., David M., Lincoln T. (2016). Piezoelectric Compared with Conventional Rotary Osteotomy for the Prevention of Postoperative Sequelae and Complications after Surgical Extraction of Mandibular Third Molars: A Systematic Review and Meta-Analysis. Br. J. Oral Maxillofac. Surg..

[127] Rullo R., Addabbo F., Papaccio G., D’Aquino R., Festa V.M. (2013). Piezoelectric Device vs. Conventional Rotative Instruments in Impacted Third Molar Surgery: Relationships between Surgical Difficulty and Postoperative Pain with Histological Evaluations. J. Craniomaxillofac. Surg..

[128] Inchingolo A.D., Malcangi G., Ceci S., Patano A., Corriero A., Vimercati L., Azzollini D., Marinelli G., Coloccia G., Piras F. (2022). Effectiveness of SARS-CoV-2 Vaccines for Short- and Long-Term Immunity: A General Overview for the Pandemic Contrast. Int. J. Mol. Sci..

[129] Sohn D.-S., Ahn M.-R., Lee W.-H., Yeo D.-S., Lim S.-Y. (2007). Piezoelectric Osteotomy for Intraoral Harvesting of Bone Blocks. Int. J. Periodontics Restor. Dent..

[130] Gao Y., Lin Z., Rodella L.F., Buffoli B., Wu X., Zhou Y. (2014). Piezoelectric Ultrasonic Bone Surgery System in the Extraction Surgery of Supernumerary Teeth. J. Craniomaxillofac. Surg..

[131] Jiang Q., Qiu Y., Yang C., Yang J., Chen M., Zhang Z. (2015). Piezoelectric Versus Conventional Rotary Techniques for Impacted Third Molar Extraction: A Meta-Analysis of Randomized Controlled Trials. Medicine.

[132] Pereira C.C.S., Gealh W.C., Meorin-Nogueira L., Garcia-Júnior I.R., Okamoto R. (2014). Piezosurgery Applied to Implant Dentistry: Clinical and Biological Aspects. J. Oral Implantol..

[133] Gadre P., Singh D., Gadre K., Khan I. (2016). Piezosurgery for Excision of Large Osteoid Osteoma. J. Craniofac. Surg..

[134] Ge J., Yang C., Zheng J., Qian W. (2016). Piezosurgery for the Lingual Split Technique in Lingual Positioned Impacted Mandibular Third Molar Removal: A Retrospective Study. Medicine.

[135] Piersanti L., Dilorenzo M., Monaco G., Marchetti C. (2014). Piezosurgery or Conventional Rotatory Instruments for Inferior Third Molar Extractions?. J. Oral Maxillofac. Surg..

[136] Pavlíková G., Foltán R., Horká M., Hanzelka T., Borunská H., Sedý J. (2011). Piezosurgery in Oral and Maxillofacial Surgery. Int. J. Oral Maxillofac. Surg..

[137] McGuire C., Boudreau C., Prabhu N., Hong P., Bezuhly M. (2022). Piezosurgery versus Conventional Cutting Techniques in Craniofacial Surgery: A Systematic Review and Meta-Analysis. Plast. Reconstr. Surg..

[138] Mirza A.A., Alandejani T.A., Al-Sayed A.A. (2020). Piezosurgery versus Conventional Osteotomy in Rhinoplasty: A Systematic Review and Meta-Analysis. Laryngoscope.

[139] Inchingolo A.M., Malcangi G., Piras F., Palmieri G., Settanni V., Riccaldo L., Morolla R., Buongiorno S., Ruvo E., Inchingolo A.D. (2023). Precision Medicine on the Effects of Microbiota on Head–Neck Diseases and Biomarkers Diagnosis. J. Pers. Med..

[140] Yang L., Chen Y., Fang W. (2023). Piezosurgery versus Conventional Osteotomy: A Randomized Clinical Trial on Pain and Anxiety in Children with Unerupted Mandibular Third Molars. Clin. Oral Investig..

[141] Bhati B., Kukreja P., Kumar S., Rathi V.C., Singh K., Bansal S. (2017). Piezosurgery versus Rotatory Osteotomy in Mandibular Impacted Third Molar Extraction. Ann. Maxillofac. Surg..

[142] Liu J., Hua C., Pan J., Han B., Tang X. (2018). Piezosurgery vs Conventional Rotary Instrument in the Third Molar Surgery: A Systematic Review and Meta-Analysis of Randomized Controlled Trials. J. Dent. Sci..

[143] Kesler G., Shvero D.K., Tov Y.S., Romanos G. (2011). Platelet Derived Growth Factor Secretion and Bone Healing after Er:YAG Laser Bone Irradiation. J. Oral Implantol..

[144] Civak T., Ustun T., Yilmaz H.N., Gursoy B. (2021). Postoperative Evaluation of Er:YAG Laser, Piezosurgery, and Rotary Systems Used for Osteotomy in Mandibular Third-Molar Extractions. J. Craniomaxillofac. Surg..

[145] Inchingolo A.D., Inchingolo A.M., Piras F., Malcangi G., Patano A., Di Pede C., Netti A., Ciocia A.M., Corriero A., Semjonova A. (2022). A Systematic Review of Positional Plagiocephaly Prevention Methods for Patients in Development. Appl. Sci..

[146] Kondo S., Okada Y., Iseki H., Hori T., Takakura K., Kobayashi A., Nagata H. (2000). Thermological Study of Drilling Bone Tissue with a High-Speed Drill. Neurosurgery.

[147] Mozzati M., Gallesio G., Russo A., Staiti G., Mortellaro C. (2014). Third-Molar Extraction with Ultrasound Bone Surgery: A Case-Control Study. J. Craniofac. Surg..

[148] Gelet A., Chapelon J.Y., Bouvier R., Rouvière O., Lyonnet D., Dubernard J.M. (2001). Transrectal High Intensity Focused Ultrasound for the Treatment of Localized Prostate Cancer: Factors Influencing the Outcome. Eur. Urol..

[149] Sortino F., Pedullà E., Masoli V. (2008). The Piezoelectric and Rotatory Osteotomy Technique in Impacted Third Molar Surgery: Comparison of Postoperative Recovery. J. Oral Maxillofac. Surg..

[150] Lieberman J.R., Daluiski A., Einhorn T.A. (2002). The Role of Growth Factors in the Repair of Bone. Biology and Clinical Applications. J. Bone Joint. Surg. Am..

[151] Rahnama M., Czupkałło L., Czajkowski L., Grasza J., Wallner J. (2013). The Use of Piezosurgery as an Alternative Method of Minimally Invasive Surgery in the Authors’ Experience. Videosurgery Other Miniinvasive Tech..

[152] Mehrabi M., Allen J.M., Roser S.M. (2007). Therapeutic Agents in Perioperative Third Molar Surgical Procedures. Oral Maxillofac. Surg. Clin. N. Am..

[153] Benington I.C., Biagioni P.A., Briggs J., Sheridan S., Lamey P.-J. (2002). Thermal Changes Observed at Implant Sites during Internal and External Irrigation. Clin. Oral Implant. Res..

[154] Kerawala C.J., Martin I.C., Allan W., Williams E.D. (1999). The Effects of Operator Technique and Bur Design on Temperature during Osseous Preparation for Osteosynthesis Self-Tapping Screws. Oral Surg. Oral Med. Oral Pathol. Oral Radiol. Endod..

[155] Horton J.E., Tarpley T.M., Wood L.D. (1975). The Healing of Surgical Defects in Alveolar Bone Produced with Ultrasonic Instrumentation, Chisel, and Rotary Bur. Oral Surg. Oral Med. Oral Pathol..

[156] Ruvo A.T., Shugars D.A., White R.P., Phillips C. (2005). The Impact of Delayed Clinical Healing after Third Molar Surgery on Health-Related Quality-of-Life Outcomes. J. Oral Maxillofac. Surg..

[157] Güven O., Keskin A., Akal U.K. (2000). The Incidence of Cysts and Tumors around Impacted Third Molars. Int. J. Oral Maxillofac. Surg..

[158] Ercoli C., Funkenbusch P.D., Lee H.-J., Moss M.E., Graser G.N. (2004). The Influence of Drill Wear on Cutting Efficiency and Heat Production during Osteotomy Preparation for Dental Implants: A Study of Drill Durability. Int. J. Oral Maxillofac. Implant..

[159] Laforgia A., Inchingolo A.D., Piras F., Colonna V., Giorgio R.V., Carone C., Rapone B., Malcangi G., Inchingolo A.M., Inchingolo F. (2024). Therapeutic Strategies and Genetic Implications for Periodontal Disease Management: A Systematic Review. Int. J. Mol. Sci..

[160] Bui C.H., Seldin E.B., Dodson T.B. (2003). Types, Frequencies, and Risk Factors for Complications after Third Molar Extraction. J. Oral Maxillofac. Surg..

[161] González-García A., Diniz-Freitas M., Somoza-Martín M., García-García A. (2009). Ultrasonic Osteotomy in Oral Surgery and Implantology. Oral Surg. Oral Med. Oral Pathol. Oral Radiol. Endod..

[162] Beziat J.-L., Bera J.-C., Lavandier B., Gleizal A. (2007). Ultrasonic Osteotomy as a New Technique in Craniomaxillofacial Surgery. Int. J. Oral Maxillofac. Surg..

[163] Piconi C., Maccauro G. (1999). Zirconia as a Ceramic Biomaterial. Biomaterials.

[164] Patano A., Cardarelli F., Montenegro V., Ceci S., Inchingolo A.D., Semjonova A., Palmieri G., Di Pede C., Mancini A., Maggiore M.E. (2022). Early Functional Orthodontic Treatment of Bad Oral Habits with AMCOP^®^ Bio-Activators. J. Biol. Regul. Homeost. Agents.

[165] Yacker M.J., Klein M. (1996). The Effect of Irrigation on Osteotomy Depth and Bur Diameter. Int. J. Oral Maxillofac. Implant..

[166] Stelzle F., Frenkel C., Riemann M., Knipfer C., Stockmann P., Nkenke E. (2014). The Effect of Load on Heat Production, Thermal Effects and Expenditure of Time during Implant Site Preparation—An Experimental Ex Vivo Comparison between Piezosurgery and Conventional Drilling. Clin. Oral Implant. Res..

[167] Peker Tekdal G., Bostanci N., Belibasakis G.N., Gürkan A. (2016). The Effect of Piezoelectric Surgery Implant Osteotomy on Radiological and Molecular Parameters of Peri-Implant Crestal Bone Loss: A Randomized, Controlled, Split-Mouth Trial. Clin. Oral Implant. Res..

[168] Toscano N.J., Holtzclaw D., Rosen P.S. (2010). The Effect of Piezoelectric Use on Open Sinus Lift Perforation: A Retrospective Evaluation of 56 Consecutively Treated Cases from Private Practices. J. Periodontol..

[169] Røynesdal A.K., Björnland T., Barkvoll P., Haanaes H.R. (1993). The Effect of Soft-Laser Application on Postoperative Pain and Swelling. A Double-Blind, Crossover Study. Int. J. Oral Maxillofac. Surg..

[170] Lamazza L., Garreffa G., Laurito D., Lollobrigida M., Palmieri L., De Biase A. (2016). Temperature Values Variability in Piezoelectric Implant Site Preparation: Differences between Cortical and Corticocancellous Bovine Bone. Biomed. Res. Int..

[171] Zhao J., Huang C. (2014). The advanced techniques of dentoalveolar surgery. Hua Xi Kou Qiang Yi Xue Za Zhi.

[172] Marsell R., Einhorn T.A. (2011). The Biology of Fracture Healing. Injury.

[173] Nordström R.E., Nordström R.M. (1987). The Effect of Corticosteroids on Postoperative Edema. Plast. Reconstr. Surg..

[174] Eriksson A.R., Albrektsson T., Albrektsson B. (1984). Heat Caused by Drilling Cortical Bone. Temperature Measured in Vivo in Patients and Animals. Acta Orthop. Scand..

